# “With Enthusiasm and Energy throughout the Day”: Promoting a Physically Active Lifestyle in People with Intellectual Disability by Using a Participatory Approach

**DOI:** 10.3390/ijerph182312329

**Published:** 2021-11-24

**Authors:** Antonia Mauro, Dirk Bruland, Änne-Dörte Latteck

**Affiliations:** FH Bielefeld, University of Applied Sciences, Interaktion 1, 33619 Bielefeld, Germany; antonia.mauro@fh-bielefeld.de (A.M.); dirk.bruland@fh-bielefeld.de (D.B.)

**Keywords:** people with intellectual disabilities, physical activity, qualitative interviews, participatory approach, target group specific concepts

## Abstract

People with intellectual disabilities have a comparatively low level of physical activity and are affected by associated numerous impairments and diseases. However, target group specific interventions and concepts aiming at sustainable physical activity-related behavior change in everyday life are rare and the needs and perspectives of the target group have hardly been considered so far. Therefore, a target-group oriented intervention was developed. The research and developmental process was characterized by a participatory approach, involving people with intellectual disabilities throughout the whole process. For example, the interview guideline, design of the interview situation and ways of communicating were developed in a participatory manner. Twenty-four qualitative interviews with people with intellectual disabilities were conducted to explore individual physical activity-related experiences and strategies. Individual and contextual influences on physical activity were identified. Interview results were used to conceptualize an intervention that aims at promoting physical activity in the everyday life of people with intellectual disabilities by strengthening their self-management strategies. The intervention was tested in practice and modified based on communicative validation methods. Good acceptance in the long term is also expected, as the intervention takes place in people’s living environment, is socially embedded and builds upon the needs of the target group.

## 1. Introduction

Physical inactivity is a huge global public health problem, as it causes both morbidity and mortality and is associated with a major economic burden [1,2,3,4]. Globally, 1 in 4 adults do not meet the global recommendations for physical activity which are set by the World Health Organization (WHO). The WHO recommends 150 min of moderate-intensity activity per week or equivalent [5]. People with intellectual disabilities show significant lower levels of physical activity compared to the general population [6,7] and are probably insufficiently active to achieve health benefits [7]. According to the World Health Organization [8], intellectual disability is understood as the reduced ability to understand new or complex information and to learn and apply new skills. It is associated with reduced communication, cognitive, literacy, and self-awareness skills [9]. In 2008, Stanish [10] noted specific barriers for people with intellectual disabilities, which are still up to date: “(1) limited options and choices for leisure in the community for people with intellectual disabilities, (2) limited financial resources required for services like transportation and staff, (3) staffing ratios that precluded the adults with intellectual disabilities from having the support that they required to engage in an activity, (4) limited financial resources of people with intellectual disabilities required for program/facility fees and transportation, and (5) unclear policy guidelines for residential and day program service provision. However, being physically active is associated with positive health effects, which also holds true for people with intellectual disabilities [11]. Regular engagement in physical activity contributes to the prevention and treatment of non-communicable diseases, hypertension, overweight and obesity. Further, mental health, quality of life and well-being are positively affected by physical activity [5].

A literature and database review of effective factors for health promotion and prevention approaches for people with disabilities [12] states that interventions that aim to promote physical activity should particularly facilitate long-term physical activity behavior. Concepts should be low-threshold and provide easy access, and they should be integrated into everyday life of the target group. To promote individual health competencies and enable people to make self-determined and autonomous decisions and actions, it is recommended to use multimodal and interdisciplinary strategies and build on educational intervention components. Applying participatory approaches is also an essential factor. A scoping review investigating the state of the art in physical activity interventions for people with intellectual disabilities [13] reveals that only a few of the existing interventions are integrated into the daily living environment of the target group. Further, it confirms Willems et al. [14], who criticize existing interventions for people with intellectual disabilities which usually do not focus on sustainable lifestyle change and do not sufficiently consider the perspective of the users. The involved studies are also too heterogeneous to draw scientifically evident conclusions for successful interventions [14,15].

The research project “Promoting physical activity and physical activity related competencies in people with intellectual disabilities” was part of the research network “User-oriented care: Promoting Health in Chronic Illness and the Need for Care”. The cooperation partner in practice was an integration assistance institution (Lebenshilfe Brakel). The project was a third-party funded study supported by the Stiftung Wohlfahrtspflege (funding period: 01.06.2018–31.03.2021). The objective was to conceptualize and test an intervention that meets the needs of people with intellectual disability and enables them to initiate and maintain a physically active lifestyle by strengthening physical activity related health competencies.

**Figure 1:**
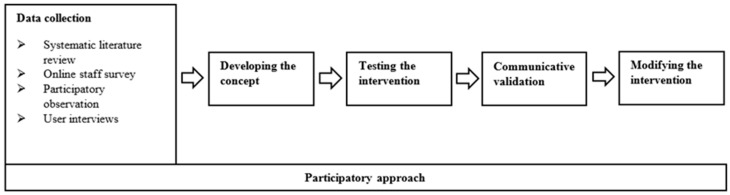
Development process of the physical activity promoting intervention for people with intellectual disabilities.

According to the aim of the special issue, this contribution describes the developmental process in chronological order (see Figure 1), taking into account the perspective of users via a participative approach throughout the whole process. In addition, how implications for a target group-specific concept were derived is described on the basis of the perspectives of the users. Regarding the developmental process, the focus of the discussion lies on the challenges and dynamics as well as the advantages of the following developmental procedure.

## 2. Participative Approach

The research process is the groundwork for the development of an intervention in this project. It was assumed that health promotion concepts will find greater acceptance in the user group if their knowledge, needs, and specific living environments are taken into account in the whole research process. The goal was to let the users participate as much as possible, in every step of the research project. The approach was oriented to the definition of Participatory Health Research (PHR) of the International Collaboration for Participatory Health Research, as follows:

“The goal of PHR is to maximize the participation of those whose life or work is the subject of the research in all stages of the research process. Such participation is the core, defining principle of PHR, setting this type of research apart from other approaches in the health field. Research is not done “on” people as passive subjects providing “data” but “with” them to provide relevant information for improving their lives. The entire research process is viewed as a partnership between stakeholders which may include academic researchers; professionals in the fields of health care, education and social welfare; members of civil society; policy makers and others” [16].

Since the 2000s, there has been an increase in German-speaking research projects involving people with intellectual disabilities in the research process; this took place much later than other international research [17]. Furthermore, in Germany there are special influences on research possibilities. Especially, societal developments in the last two decades have brought about great changes, above all changes in the idea of care and the special legal situation, e.g., the Federal Participation Act, which enters into force in four staggered reform stages by 2023. Therefore, the project follows the current state of knowledge for research participation of people with intellectual disabilities according to the German participatory health research network (partnet). Experts in partnet are scientists, practitioners, civil society activists and others with their own specific experience.

In the present project an expert board (consisting of one academic researcher, one non-academic co-researcher, i.e., the project coordinator of the cooperating facility, and two persons with intellectual disabilities, and a research working group (as before, but with six persons with intellectual disabilities) were established for continuous collaboration. The perspective of the target group could be continuously considered and taken into account by these two groups. For example, the target group was involved in planning and carrying out information events, defining the project title, developing interview guidelines, and modifying the intervention concept. The expert board met regularly and supported the project management in each step of the process. The research working group focused on research activities, e.g., dealt with developing and testing the interview guidelines. Members were given training beforehand in order to become familiar with scientific approaches. Participative elements are described below.

## 3. Materials and Methods

Methodological triangulation (see Figure 1) was applied, involving an online staff survey (*n* = 67), a document analysis, participating observations (*n* = 3) and target-oriented problem-centered qualitative interviews with people with intellectual disabilities (*n* = 24; inclusion criteria: mild or moderate intellectual disability). The developed intervention concept was tested in practice. In this context, communicative validation methods were used in order to obtain information about practicability and acceptance of the intervention.

It is important to note that there has been a shift towards discussing disability in terms of functioning via the International Classification of Functioning, Disability and Health (ICF). It is therefore important to point out that the selection was made according to clinical diagnosis of cognitive levels. The reason for this is that in practice at the time of the interviews the International Classification of Functioning, Disability and Health had just been introduced by law in Germany and was therefore not yet practicable for our cooperation partner.

Due to the focus of this article on the perspectives of the target group, only the approach to user interviews and communicative validation is described here.

### 3.1. Interviews

#### 3.1.1. Participant Recruitment

The cooperating setting was an integration assistance institution, including in- and outpatient housing (for further demographics see Table 1). Staff, specifically the project coordinator, was essential in recruiting the interview participants when addressing these potentially. Due to the established expert board and research working group and due to information events that were planned and carried out along with these groups, the project was already known among residents, which supported the recruitment process via suggestions about suitable interview partners and publicity about the importance of the project. Informed consent of the interviewees themselves and, if necessary, of a legal guardian was obtained. The process was reviewed by the Ethics Committee of Bielefeld University (No. 2018-215-S) according to the ethical guidelines of the German Psychological Society and the Professional Association of German Psychologists and found to be ethically safe. A prerequisite for participation in the interviews was sufficient cognitive ability to concentrate and communicate. This was determined by staff of the institutions, who knew the subjects well and were able to estimate if they could be eligible for an interview situation.

#### 3.1.2. Interview Conduct and Analysis

User interviews were carried out between February and April 2019. The interviews aimed at exploring individual physical activity-related knowledge, experiences and strategies as well as individual requirements for the intervention concept. These were analyzed by means of content analysis [18] using MAXQDA software. The category system was inductively built, discussed, and revised by three researchers independently of each other.

#### 3.1.3. Participatory Elements within Design of the Interview Guideline and the Situation Itself

The participatory part was realized [19,20] as follows: The interview guideline was developed in collaboration with the research working group. Thus, relevant topics and preferred ways of communication of people with intellectual disabilities could be taken into account. In detail, aspects of language (using easy German) and illustration, using illustrative materials such as pictures and anatomy models, were considered and prepared for individual needs. The interview guideline was pre-tested by the academic researcher (author DB) for comprehensibility and feasibility within the research working group, consisting of six people with intellectual disabilities. Further, the interview situation was prepared and designed in a target group-sensitive manner. For example, a leaflet, asking simple questions concerning personal characteristics (e.g., name, hobbies, preferences and dislikes at work and in leisure time) of the interviewer and the interviewee was introduced at the beginning of the interview situation in order to facilitate familiarity with the situation itself and the interviewer.

### 3.2. Test of the Intervention and Communicative Validation

The intervention was developed based on described data collections and was tested from 1st July 2020 to 30th September 2020 starting with *n* = 33 participants. Communicative validation was used to obtain information about the applicability of the intervention. Users and their buddies (buddies: see below) were asked to answer questions on topics of duration, comprehensibility, manual design and individual effects. Statements (*n* = 127) were structured in terms of content and prepared for communicative validation. Here, communicative validation means that the researcher’s interpretation of the results was presented to the target persons for discussion [21].

The participatory part was realized in the context of group discussions. The group discussions took place with the directors of the living areas, with two groups of buddies (four buddies in each group), and with the expert board. Due to Covid19 restrictions, discussions were conducted online with the expert board. Planned discussions with the research working group had to be cancelled due to the situation and to the low levels of digital literacy in the institution. Subsequently, the concept was modified.

## 4. Interview Results

The following section deals with the implications for intervention development, based on the results of the user interviews and effective target group oriented approaches to health promotion and prevention. The numbers in brackets indicate the interview number and the position of the citation within the interview transcript.

For orientation: a short structure of the results section is divided into brief sections in order to illustrate the developmental process, built upon different steps:

- Interview results and implications for physical activity promoting concepts
- Conceptualization of the intervention
  ⚬ Implications of the user interviews
  ⚬ Considering the theoretical background
- The initial intervention concept
- Testing and modifying the intervention
- Content and structure of the final user manual

### Interview Results and Implications for Physical Activity Promoting Concepts

Here, categories that were formed within the structured interview analysis are presented. Interview quotes were translated from German into English; an attempt was made to reproduce the statements correctly. To sum up, most participants know about the importance of being physically active and report good motivation levels. However, multiple barriers exist. Implications that were made in order to develop the intervention concept are described in each of the categories (see below and Table 2).

Individual factors:

Physical and cognitive conditions: Barriers to physical activity are often due to physical and psychological conditions. Most participants mention various movement limitations, e.g., spasticity, pain, risk (and/or fear) of falling, limb and cardiovascular problems (author note: the medical language here is used by interviewees themselves) and are in need for support when planning to be and/or being active.

“I am always dependent on someone coming along. I can’t go out there alone anyway, (…). That’s not possible.” [15] (p. 74) (numbers in brackets indicate the interview number and the position of the citation within the interview transcript).

This means that interventions have to be adaptable to an individual’s needs. A support person is also likely to be needed throughout the whole intervention process. In order to address various cognitive abilities and individual preferences for communicating, different ways of providing information are suggested, e.g., interactive parts, reading parts, offering creative ways of obtaining knowledge and gaining experiences.

Physical activity related knowledge: Most participants know about the importance of being physically active and specific goals can be formulated. However, depths of understanding about the links between physical activity and health and the ability to differentiate and apply this to one’s own person and situation differ. Being physically active is often associated with doing exhaustive sports. Leisure time, occupational, domestic and commuting physical activity are not mentioned and not regarded as important elements of physical activity.

“(…) And there are only a few types of sports coming to my mind, because I don’t know what I could do due to my disability.” [8] (p. 53).

Hence, it is proposed that interventions provide basic health and physical activity related knowledge that can be adapted to the needs and interests of the individuals. A broader understanding of physical activity should also be delivered.

Physical activity related experiences and associations: Participants report both positive and negative physical activity related experiences. Negative experiences refer to past events such as injuries and falls or to feelings of physical limitations and pain. Good experiences refer to feelings of joy, enjoyment, relaxation, well-being.

“Interviewer (I): And then did you hurt yourself a lot when you fell? Respondent (R): Yes. I: I’m sorry about that. (…) Are you still afraid to walk alone too? R: Afraid to walk alone, yes.” [4] (pp. 174–179).

“I: When you move, why do you do it? R: Yes, because it makes me feel good.” [2] (pp. 298–301).

Physical activity promoting interventions have to address such experiences. This can either be built upon existing positive associations, or new experiences have to be initiated.

Self-management strategies (strategies that can be used by individuals to plan and implement a specific behaviour (e.g., [22].) for being physically active: There is a lack of self-management strategies, impeding the implementation of physical activity into everyday life. Only a few respondents reported strategies that they apply in order to deal with internal and/or external barriers to being physically active.

“Yeah, I’ve been trying to back out, too. But then I think, just do it, it’s good for you, then you’re in a completely different mood when you get home.” [22] (pp. 155–156).

Interventions should therefore provide tools that help users to strengthen their skills for successfully planning and implementing physical activity.

Contextual factors:

Social support: Social contact can be desired or perceived as restrictive. Support from other people, e.g., in the form of companionship or supervision, is mentioned as helpful and often necessary when exercising. People, e.g., from private or professional contexts, can provide safety, can motivate and/or make it fun. In contrast, some participants prefer being physically active alone, for different reasons. For example, social fears can exist, or tranquility is enjoyed, when being alone.

“I enjoy that. When you’re there with others, you can have a good laugh.” [22] (p. 268).

“I have peace and it’s quiet and I can relax on big walks. Alone.” [14] (p. 208).

This means that interventions have to consider individual preferences and experience based attitudes.

Physical activity offers: Many existing physical activity opportunities were mentioned by respondents. However, costs for sports offers or for public facilities can be a barrier. In addition, it is reported that staff capacities are limited or movement-specific qualifications of the staff are lacking.

“No, swimming not so often now, because the supervisors are not educated as lifeguards.” [13] (p. 201).

This means that access barriers referring to financial or staff related constraints have to be considered. Low-threshold access should be promoted.

Tight day schedule: The interviewees are subject to a very structured everyday life. Existing physical activity offers are more or less integrated into this structure, but are sometimes subject to obstacles if they do not fit into the structure in terms of time or associated factors (e.g., darkness, being tired, being visited, having other stuff to do).

R: “(…) And half past five walking starts. (…) Then I’m still tired. Because I was eight hours at work.” I: “Yes. And if you would say, yeah, walking at six?” R: “(…) At six, we made a plan for morning, lunch, supper. That we have to be ready at half past six. And clean up at seven in peace.” [23] (pp. 58–161).

Interventions should consider that everyday life physical activity is likely to be integrated more easily into a strongly structured everyday life than movement offers such as exercise courses.

Barrier free environments: Environmental conditions that are not suitable for people with disabilities, such as roads with heavy traffic or uneven surfaces, are often described as barriers to physical activity. Participants report that walking independently (possibly with aids) then becomes more difficult and makes support from other people necessary. Sports facilities are described as either easily accessible or not.

R: “(…) When I go for a walk, I take my walker. (…) I mean I have quite enormous problems.” (The town center in the place of residence is difficult to pass due to cobblestones) [14] (pp. 41–45).

A non-barrier-free environment that hinders people with disabilities from being physically active is a fundamental problem and has to be considered when conceptualizing interventions.

Weather conditions: Weather conditions are mentioned by all respondents and very frequently. They can be both a facilitating and hindering factor. The way this is dealt with varies. In some cases, bad weather conditions, for example, cold weather in winter, directly equate to less active behavior. Occasionally, it is reported that weather-appropriate clothing is chosen in order to be physically active outdoors despite poor conditions. Thus, weather conditions have a major impact on physical activity behavior.

I: “Are there days when you exercise more than other days?” R: “In summer. In winter, I rarely move.” [6] (pp. 111–112).

A barrier such as bad weather can be addressed by promoting self-management strategies.

Short summary: Individual (physical and psychological), social and environmental factors have an influence on the physical activity behavior of the interviewed persons. The lack of self-regulatory strategies, e.g., difficulties in implementing physical activity, is assumed to be a central obstacle to physical activity behavior. The aim of the intervention therefore is to enable the users to strengthen their self-management strategies in order to learn to cope with internal and external barriers. The results were discussed within the analyzing process with the research working group [23].

## 5. Conceptualization of the Intervention

The conceptualization of the intervention was based on the described implications derived from the user interviews, as well as on theoretical background, as illustrated below. Revisions within the development process are also presented, which were made after testing the intervention in practice.

### 5.1. Implications from the User Interviews

In order to enable users to cope with internal and external barriers, the intervention concept deals with strengthening individual self-management strategies. Users shall acquire knowledge (in theory and practice) about physical activity and health, especially linked to the specific person. For example, users are encouraged to learn about their body, their individual needs and physical and mental capacities. They are supposed to learn more about their personal physical activity related goals and how these goals can be achieved. This is about planning physical activity and putting it into action. Users will be enabled to identify barriers to being physically active and they can learn to find out about possible strategies to overcome barriers. This could be built upon existing knowledge, inferred from the interviews. Interview results also showed that users are in need of support when dealing with new inputs and/or being physically active. Moreover, interviews showed a great heterogeneity among users in terms of cognitive and physical abilities, which was also considered by inventing the role of a physical activity buddy (see below).

### 5.2. Considering the Theoretical Background

Theoretically, the conceptualization of the intervention was based on special impact and key factors, described below. For more details concerning the background see [24,25].

Impact factors:

Three impact factors for effective interventions were described within the literature and database review of health promotion and prevention approaches for people with disabilities and the evaluation of available evidence [12]: multimodality, participation and living environment. How these factors are considered in the intervention concept is described below.

Multi-modality: Different levels of learning are addressed: this is about experiencing physical activity in a positive way, having an interactive exchange with supporting persons and/or buddies. In addition, information is provided in easy-to-understand language that is tailored to the target group and can be read with or without help.

Participation: By using a participatory approach, the needs of potential users can be identified. From the beginning of the project, potential users were involved. A planning group was established consisting of the project coordinator of the practice partner and two people with intellectual disabilities. This group was a council of experts that was consulted at each stage of the project. A research group, consisting of the project coordinator of the practice partner and six people with intellectual disabilities, accompanied the research tasks.

Living environment: The intervention should be low-threshold and take place or be easily integrated into the living environment of the users. Here, opportunities for being active in everyday life can be identified. In addition, staff and management are introduced to the topic by providing information on the design of (social) living environments conducive to physical activity.

Aspects of promoting self-management/self-efficacy were added according to the project aim [15]: the intervention focuses on the promotion of individual physical activity-related competencies. The goal is to enable participants to engage in physical activity in a self-determined way and over the long term. This means that participants should be enabled to build physical activity-related goals and to plan and implement physical activity. In addition, participants should be able to recognize internal and external obstacles and find and apply coping strategies.

Key factors:

Nutsch et al. [13] describe key factors of effective interventions that aim to promote physical activity in the everyday life of people with intellectual disability: (a) nearly all successful interventions were multi-component interventions and applied on different levels; (b) participation of intervention users and pre-intervention needs assessment; (c) relevance to everyday life; (d) interventions are related to individuals with intellectual disabilities and address the whole organization including the caregivers; (e) using a combination of education sessions and practical training. Target group-oriented educational materials or technical devices should be used. Further, successful physical activity interventions are theory-based and formulate intervention goals.

Underlying model:

Based on the results of the interviews the intervention concept was oriented towards the model of physical activity-related health competence, which contains three major competences: movement, control and self-regulation competences. The advantage of this model is that it not only focuses on increasing physical activity through what is offered, but addresses the person by considering physical prerequisites, fitness, knowledge, motivation, and strategies for action [26].

### 5.3. The Initial Intervention Concept

The core of the intervention is the user manual. The manual is to be understood as a tool that can be used for continuous reflection in order to maintain or, if necessary, adapt behavior when obstacles arise. The intervention comprises a manual for users, buddies and staff/the organization. Another central element of the intervention is the physical activity buddy. Both elements are described below.

The first user manual:

The initial user manual consisted of the following ten units:

Physical Activity Buddy; Physical Activity and Health; Physical Activity Quiz; How much should I do? Physical Activity Passport; Wishes and Goals; Observation Sheet, Achieving Goals; How does it Work?; Physical Activity and Nutrition. The structure and content of the user manual were modified after the test of the intervention in practice (described below).

The physical activity buddy:

The research working group gave hints in the developmental process [27] for the desired support, according to individual needs, of support persons. Physical activity buddies are persons who are familiar to the user and who accompany him/her during the process and provide resource-oriented support. These persons are ideally selected by the users themselves at the beginning of the intervention. They can be friends, family members or facility staff, for example caregivers. At the beginning of the intervention, users and their buddies make a contract with each other that creates a commitment for both sides. The task of the buddy is to work on the manual together with the user, i.e., to read it together, to talk about it and to contribute further individual-specific ideas. They are equipped with an additional accompanying manual, which provides user-relevant knowledge and a selection of exercises analogous to the units of the user manual. Buddies have an essential role in that they are the ones who can flexibly adapt ideas and instructions to the specific needs, individual resources and environmental conditions of the user.

## 6. Testing and Modifying the Intervention

### 6.1. Testing the Intervention

Feedback of 28 users (five dropouts) and their buddies while testing the intervention in practice were evaluated (see Table 3).

Two group discussions with four physical activity buddies each were conducted to discuss the results. As already mentioned in the methods section, the expert board was also involved in the process of revising the results. All groups confirmed the great heterogeneity of individual and environmental prerequisites, examples of which can be seen in the contradictory statements (see Table 3). Content and structure of the user manual were evaluated in a range from far too simple to far too complex. In order to better address this heterogeneity, the possibility and necessity of flexibly adapting the manual to the individual’s needs was emphasized and the manual was revised accordingly: the units “Physical Activity Quiz” and “Physical Activity and Nutrition” were cancelled due to the feedback. All participants wanted more details that were not possible to implement without further increasing the complexity. The structure of the user manual was modified in order to reduce complexity and to achieve a greater flexibility. Furthermore, a second manual entailing a higher language level was produced in order to meet the different needs and abilities. The manual was redesigned to make it more attractive for users. The importance of a support person was highlighted.

### 6.2. Content and Structure of the Final User Manual

Modules (see Table 4): The user manual consists of four modules and a total of twelve units, which are supported by physical activity buddies. Module 1 serves as an introduction to the intervention. Here, an overview of the content and goals is given and the search for a physical activity buddy (see the following section) is initiated. The “Physical Activity Passport” is also introduced to the users. This is a personal tool that helps users to develop orientation in the process from the beginning on and to record important physical activity-related information. Module 2 is about providing basic information on physical activity and health. It aims to promote knowledge-related competencies, motivation for physical activity, and positive physical activity-related associations. This is implemented through information written in simple language and containing motivating and illustrative picture elements. Exercises for experiencing physical activity are also essential, including suggestions for exchange and reflection with their physical activity buddy. Exercise example: it was suggested to hold up a balloon and reflect on the experiences and associations: e.g., was this already a physical activity? Was it fun? Was it hard? The learning experience can be that physical activity goes beyond exercise alone and can also be playful and easy. Barriers to associated exhausting sports will therefore be met. Module 3 deals with finding and formulating personal physical activity related goals and planning physical activity. In other words, it is about initiating and maintaining goal-oriented physical activity. Tools are used to enable action implementation. The planned activity can be recorded in a personal movement overview and be planned using a weekly schedule. An observation sheet provides support for reflecting on one’s own behavior and identifying and managing possible barriers. Module 4 aims to create a routine and promote self-management in the long term.

## 7. Discussion

The research project aimed to develop and test an intervention concept in a participatory manner that promotes physical activity by strengthening individual physical activity related skills in people with intellectual disabilities. According to the presented developmental process, challenges and dynamics as well as the advantages of the chosen procedure will be discussed.

### 7.1. Comparisons with Other Projects

A comparison with other interventions seems to be difficult because they pursue very different goals and include a variety of different approaches [28,29]. The review by Latteck [12] concludes that effective interventions include three approaches: (a) Multimodal, (b) Participatory, (c) Living environment orientation. The conceptualization of the presented intervention is based on these criteria and on the results of the literature review which has been conducted at the beginning of the research project [15]. Here, only 11 interventions could be included that are integrated into the living environment and address the promotion of physical activity (and self-management) [13]. While almost all interventions included multimodal approaches, user involvement in intervention development was described in only four studies. Of these, two addressed staff [30,31] and only two addressed users themselves [32,33]. In Bodde et al. [32], as an example, only the users were interviewed after the intervention development to discuss two units of the intervention. The participatory approach of the present intervention, which refers to the entire research process, can thus be described as innovative. Further, almost all interventions relied on group offers. Our findings from the interviews and the few individual offerings described [34] lead to the assumption that motivation and action implementation (volition) require a thorough addressing of personal needs and goals.

### 7.2. Challenges

The participatory approach posed various challenges to the project, increasing the complexity of the process. This begins with the selection of the co-researchers (people with intellectual disabilities) for the participatory groups. On the one hand, all persons should be given equal opportunities to participate; on the other hand, participatory work requires more time, e.g., for longer decision-making processes and the preparation of information appropriate to the target group. This is a major field of tension in typical funded project durations [35]. In our project, we therefore decided to target individuals for the planning group (which met more frequently) who had certain prerequisites in mobility (independent use of public transportation), language skills, and ability to concentrate. The selection of the eligible persons was made in consultation between the project coordinator of Lebenshilfe Brakel and the scientist of the FH Bielefeld, who got to know persons in the field phase. For the research group, which did not meet so frequently and within which the heterogeneity of the target group was to be reflected, the selection was left to the management of the residential areas. In addition, the user survey and the target group-specific preparation required a high level of methodological competence by the researchers. Known methods had to be adapted and further developed, and the support of facility staff was often necessary to explain information.

Despite the amount of work and time involved, the participatory work has proven to be highly rewarding. The participatory approach ultimately had an extremely positive effect on the development of the intervention. It is geared towards the needs of the users and the acceptance of the intervention appears to be very high according to the results of the test phase and communicative validation.

### 7.3. Dynamics

With the participatory approach and results gained from asking potential users themselves via interviews, we unleashed a different dynamic in the discussion about the intervention development. The focus of the project discussion was on how health related information is received and how health-related decisions are made. Here there seems to be a major potential for developing interventions, which is usually not mentioned in article describing this process [13].

The interview results enabled the perspectives of the potential users to be taken into account. For example, control competence in the conceptualization chapter model of physical activity-related health competencies includes effect knowledge and action knowledge, with the focus on health-enhancing physical activity [26]. The control competence involves health literacy, here understood as the investigation and promotion of health information processing as a starting point to action [26]. Our interview results highlighted the importance of focusing on individual and contextual factors. Competencies and decisions (decision making) are manifested in actions and these are linked to values, attitudes, feelings and social relationships [36,37]. Therefore, when promoting health literacy, focus should not only be on the users, but the social components must also be taken into account, in particular, the living environment (culture) in terms of housing, work and leisure. It is advocated that health literacy is considered as social practice, i.e., as activities that are always embedded in specific situations and contexts and whose actual form and meaning can only be understood within these contexts [36,37]. More significant than the acquisition of health information is what people are willing and are able to put into action in their life contexts with the given information [36]. Rather, when giving people with intellectual disabilities information and exercises, the social practices that rely on the skills of communicating health information and its beliefs, values, attitudes, and the influence of social structures such as peers, family, and especially health professionals (staff) have to be taken into account, among others [37]. This is confirmed by the research project presented here. The participatory approach gives us hints regarding individual needs, desired support and what is needed from the organization. Therefore, various manuals were developed (for users, for physical activity buddies and for staff, respectively, for organizational processes).

### 7.4. Limitations

There are some limitations that have to be mentioned. Concerning the recruitment process, staff were central in assessing the interview eligibility of potential interview participants. Thus, the selection process lacked objectivity and may have led to the preclusion of other eligible and interested participants. In this way, we were able to ensure that no participants were included for whom participation would have been a disadvantage, also in view of the lack of time resources which did not allow a more comprehensive approach. Further, the heterogeneity of the target group was confirmed within our project. Therefore, design and conduction of the interviews were adapted to the individual and heterogeneous needs of the interviewees. Within the interview analysis, a high degree of interpretation was sometimes necessary in order to understand what had been said and to be able to embed it into the individual context. In addition, the response behavior seemed to be partly characterized by social desirability. Supplementary interviews with professionals could have validated the users’ statements. Here, however, the focus was on the user’s perspective and other perspectives should not be given more weight due to linguistic advantages. The user interviews were characterized by their target group-sensitive preparation and implementation. The content and structure of the guidelines, the design of the interviews and the interview situation were developed in a participatory manner with the target group which made it possible to address specific topics relevant to the target group and its living environment and to develop a concept that aims at meeting the various needs of the target group.

### 7.5. Distribution of the Intervention

When the article was written, the intervention was distributed in several ways: possible facilities were informed that will use our intervention. The intervention was presented at different conferences, within the terms of use. Finally, a manual will be published by a major publisher who has experience nationwide. A translated manual in the English language is considered as a possibility. Results of communicative validation suggest that the intervention can be well integrated into the daily work routine and that the services are affordable, leading to a good cost-benefit balance. In order to be able to make reliable statements about the effectiveness of the intervention, an evaluation is planned including cost-effectiveness for the proposal used in the community. At the moment, we are looking for funding.

Furthermore, measures that could usefully complement the intervention should be discussed. For example, the use of peer mentors was discussed during the development phase of the intervention, but adequate implementation would have exceeded the time frame of the project. This should be considered in the future, as peer mentors can reinforce a target-group-specific approach [38].

## 8. Conclusions

The aim of the research project was to develop an intervention for people with intellectual disabilities that strengthens self-management competencies for initiating and maintaining physically active behavior in everyday life. This participatory project resulted in an intervention that promotes everyday physical activity in people with intellectual disabilities through facilitating the development of knowledge and strategies of self-management (through subjective reflections on one’s own behavior, individually developed goals and barrier management). The practical manageability of the concept was confirmed by a trial phase. Due to the consideration of the user’s perspective, the intervention experienced a high acceptance. It can be assumed that the intervention will have positive effects on the users’ movement abilities (e.g., by preserving muscles, promoting coordination skills, and therefore reducing falls and the consequences of falls). In the case of existing illnesses, it can be assumed that it will contribute to a positive coping process in those who are affected. It provides caregivers with a scientifically based and practically tested intervention that takes the greatest possible account of the users’ self-determination.

## Figures and Tables

**Table 1:** Demographics of interview partners.

Sex	15 Female	9 Male
Living area	20 outpatient	4 inpatient
Age	21–68 years of age, mean age 44 years	
Work area	2 retired persons, 1 person working in regular labor market, 21 persons working in workshops for disabled persons	

**Table 2:** Overview of individual and environmental factors influencing physical activity behavior, and implications for the development of physical activity-promoting interventions.

Categories and Subcategories			Implications
Individual factors	Physical and cognitive cognitions		Diversity of cognitive and physical limitations and support needs have to be considered by offering flexible and adaptable approaches.
	Physical activity related knowledge		Basics of physical activity-related knowledge (including distinguishing types of physical activity) have to be provided.
	Psychological factors	Physical activity related experiences and associations	It should be built upon positive experiences/these should be created. Heterogeneous physical activity-related experiences and preferences should be addressed.
		Self-management strategies for being physically active	Strengthening of self-management strategies is needed for dealing with internal and external obstacles.
Contextual factors	Social factors	Social support	In many cases, support from another person is indicated and/or desired. Addressing different user needs and preferences.
	Environmental factors	Tight day structure	Exercise in everyday life should be promoted.
		Physical activity programs	Low-threshold access is needed (considering the living environment rather than creating new programs).
		Barrier-free environments	Environmental conditions in the context of individual physical conditions have to be considered.
		Weather conditions	Promotion of self-management strategies is suggested.

**Table 3:** Overview: results of testing the intervention.

Topic	Negative Feedback	Positive Feedback
Implementation process	- more information needed e.g., about the role of the Physical activity—Buddy	
Structural design (instructions, structure, materials, comprehensibility, language used, pictures)	- contract commitment - more pictures are needed - text is too complicated, too childlike	- contract commitment - visualizing is good - text is good to understand
Content (are topics covered that are considered important, adequately described, are desired topics missing?)	- more information about nutrition is wanted	- content about nutrition is good, try outs and learning practically
Feasibility of implementation (e.g., target group specific/fit for client/over-demanding, supervision necessary)	- organizational problems: chargeable?	- good explanations
Assumptions of effect & sustainability	- only with more support possible	- commitment is to observe - entering into conversation is observed - formulating and recording own goals and movements happens

**Table 4:** Overview of modules and units within the user manual.

User Manual	
Modules	Units
(1) Introduction	Introduction and overview
	1. Physical activity buddy
	2. Physical activity passport
(2) Gaining knowledge and experience	3. What is physical activity?
	4. Why is it important?
	5. How can I be active in everyday life?
	6. Physical activity and health
	7. How much should I do?
(3) Planning and being active	8. Physical activity related goals
	9. Observation sheet
(4) Reflections and routines	10. Self-reflection
	11. Meeting barriers
	12. How to proceed?

## Data Availability

Not applicable.
